# Snakebite incidence and healthcare-seeking behaviors in Eastern Province, Rwanda: A cross-sectional study

**DOI:** 10.1371/journal.pntd.0012378

**Published:** 2024-08-21

**Authors:** Dieudonne Hakizimana, Lauren E. MacDonald, Happy Tahirih Kampire, Mihigo Bonaventure, Mahlet Tadesse, Elijah Murara, Leila Dusabe, Leandre Ishema, Janna M. Schurer

**Affiliations:** 1 University of Washington, Department of Global Health, Seattle, Washington, United States of America; 2 Department of Global Health Delivery, University of Global Health Equity, Butaro, Rwanda; 3 World Health Organization Regional Office for Europe, Copenhagen, Denmark; 4 Center for One Health, University of Global Health Equity, Butaro, Rwanda; 5 Department of Infectious Disease and Global Health, Cummings School of Veterinary Medicine at Tufts University, North Grafton, Massachusetts, United States of America; The George Institute for Global Health India, INDIA

## Abstract

**Background:**

Snakebite envenoming (SBE) is a potentially life-threatening event that can lead to severe physical, mental, and economic hardships, particularly in under-resourced regions like sub-Saharan Africa. In Rwanda, there have been no epidemiological assessments of SBE to guide the Ministry of Health in its efforts to reduce the burden. This study had two main objectives: first, to estimate the incidence of snakebites across districts, and second, to describe formal versus informal healthcare seeking behaviors among snakebite victims in Eastern Province, Rwanda in 2020.

**Methodology:**

This cross-sectional study utilized a cluster sampling approach, involving Community Health Workers (CHWs) who recorded snakebite cases across seven districts. The descriptive analysis considered sampling weights, and healthcare seeking behavior was assessed based on the type of care sought as the first point of treatment.

**Findings:**

The study surveyed 390,546 individuals across 763 villages and estimated a provincial annual incidence rate of 4.3 cases per 1,000 individuals. Incidence estimates ranged from 1.1 cases per 1,000 in Nyagatare to 9.1 cases per 1,000 individuals in Bugesera and Ngoma districts. Among the 2,545 cases recorded by CHWs, three resulted in deaths. Regarding healthcare-seeking behavior, 13% of snakebite victims (143 out of 1,098) initially consulted formal care providers (CHWs, health post/center, or hospital), while 87% sought informal care (family/friends, pharmacist, or traditional healer). Approximately half of the victims (583, 53.1%) reported severe symptoms. Unsafe practices included skin cutting/burning, tourniquet application, use of black stones, and venom extraction; only 24 cases (2.2%) received anti-venom.

**Conclusions:**

This large-scale community-based assessment highlights variations in snakebite incidence between districts and confirms frequent involvement of traditional healers in management. Improving access to anti-venom and community education on the risks of ineffective practices, along with timely use of formal healthcare, are crucial. Collaboration between healthcare providers, traditional healers, community leaders, and policymakers is essential to implement targeted interventions for enhancing snakebite prevention and management strategies.

## Author summary

Snakebites can be life-threatening, especially in places like sub-Saharan Africa. In Rwanda, there haven’t been any studies to understand how many snakebites happen and where they happen. This study wanted to find out two main things: first, how many snakebites happened in different parts of the Eastern Province of Rwanda in 2020, and second, where people who got bitten by snakes went for help—to doctors or traditional healers.

The study involved 390,546 people across 763 villages. It found that each year, about 4.3 out of 1,000 people in the province get bitten by snakes, but the numbers vary in different areas. Surprisingly, only 13% of the victims initially consulted formal healthcare providers like community health workers, health centers, or hospitals. In contrast, a larger group, 87%, sought informal care from family, friends, pharmacists, or traditional healers first. About half of the people bitten by snakes experienced severe symptoms. Unfortunately, many people used unsafe methods to treat snakebites, like cutting or burning the skin, tying a tight band around the bitten area, using black stones, or trying to suck out the venom. Only a very small number of cases, just 24 out of all 1,098 victims, received anti-venom.

The study suggests we need to make sure anti-venom is easier to get and teach communities about the risks of unsafe practices and why it’s important to get formal healthcare quickly. It’s important for doctors, traditional healers, community leaders, and government officials to work together to make sure people get safe and appropriate treatment and learn how to prevent snakebites.

## Introduction

Snakebite envenoming (SBE) is one of the world’s most neglected tropical diseases (NTDs), especially in rural Sub-Saharan Africa (SSA) where access to snake anti-venom and well-equipped facilities is low [1]. Snakebites can be physically, psychologically, and economically devastating due to the risk of long-term disability or death, the high occurrence among working age individuals, and the high cost of care. Those at highest risk are generally impoverished farmers and children working in rural and remote areas [2,3]. In SSA, human cases are estimated to exceed 268,000 annually, causing a burden of 1.03 million DALYs (95% CI: 0.80–1.28 million), while the incidence of livestock cases, and consequent burden on livestock owners, has received little attention [4,5].

In Rwanda, health systems challenges for preventing and treating snakebites are not unique. Hospitals experience frequent snake anti-venom stock-outs and physicians report poor confidence in treating serious cases [6–8]. Health facilities more often stock inappropriate anti-venom than those with proven efficacy against African snakes [6,8]. Standard protocols for diagnosing and evaluating SBE, such as simple blood clotting tests, are not consistently utilized. Some healthcare providers prescribe ineffective treatments, such as blackstone, or refer patients to traditional healers [7]. As is common across SSA, SBE is often perceived as a spiritual event that physicians are unable to fully treat, causing victims to seek out traditional medicine [3,9]. No efforts have been made to evaluate traditional healers’ participation in SBE management, as occurred in Ghana [10], or to integrate them into formal strategies for burden reduction [11].

Across SSA, snakebites are under-diagnosed and under-reported [12]. In Rwanda, SBE is nationally notifiable with cases recorded in the Health Management Information System database [6]. However, only SBE victims who report to formal health facilities are documented in this system. Moreover, the proportion of victims who access formal versus informal care is not known. This research aimed to estimate geographic differences of snakebite incidence across Eastern Province in 2020 and to characterize formal versus informal care-seeking behavior among snakebite victims. Such information is needed to advocate for SBE victims at the policy level and to inform Ministry of Health goals for halving SBE related morbidity and mortality [13]. The study successfully achieved its aims by providing detailed insights into the incidence and care-seeking patterns of snakebite victims in the Eastern Province of Rwanda.

## Methods

### Ethics statement

This study protocol, including consent process, was reviewed and approved by the University of Global Health Equity (UGHE) Institutional Review Board (UGHE-IRB/2020/012). At the household level, CHWs requested permission from snakebite victims to share their contact information with the study team. Study team enumerators contacted those who agreed, and all participants provided oral informed consent (S3 Appendix) before participating in the care-seeking behavior survey. Those who did not consent were excluded.

### Study design

In order to estimate the incidence of SBE at the provincial level, we conducted a cross-sectional survey in Eastern Province. SBE cases identified through the cross-sectional survey were further interviewed to characterize care-seeking behaviors among snakebite victims.

### Study setting

Eastern Province is the largest of the five Rwanda provinces and is subdivided into seven administrative districts and 95 sectors. Its 3.6 million residents are primarily engaged in agricultural production, including staple crops and livestock rearing [14]. It contains an expansive protected wildlife area, Akagera National Park, and is bordered by Uganda, Tanzania, and Burundi. Venomous snakes include vipers (*Bitis arietans B*. *gabonica* and *B*. *nasicornis*), mambas (*Dendroaspis jamesoni* and *D*. *polylepis*), cobras (*Naja nigricollis*, *N*. *annulate*, *N*. *melanoleuca*), and asps (*Atractaspis bibronii* and *A*. *irregularis*), among others [15]. Residents access health services through a referral-based system, starting with Community Health Workers (CHWs) and progressing to health posts, health centers, and hospitals [16]. We selected Eastern Province as the study region because hospitals there reported more SBE cases than any other province in 2018 [6].

### Participants

Our study targeted all individuals residing in the Eastern Province of Rwanda in the year 2020. Table A in S1 Appendix shows the estimated population in the eastern province in 2020. According to National Institute of Statistics Rwanda census data, each district is subdivided into 12 to 15 sectors, with 2020 populations ranging from 26,962 to 40,069 people per sector [17]. Using 2012–2032 medium growth rate projections of the total population, we estimated that 3,125,692 were living in Eastern province in 2020 [17].

### Variables

#### Snakebite

To estimate the snakebite incidence, snakebite was a binary variable indicating whether a participant self-reported being bitten by a snake at least once in 2020 during the interview with the data collector.

#### Healthcare seeking behavior

This was a binary variable indicating whether a snakebite victim sought formal or informal treatment as their first point of care following the snakebite. Formal care included any level of the health system in Rwanda such as consulting a CHW, a health post, a health center, or a hospital. Informal care includes any other type of care such as self-care, traditional healers, pharmacy or any other.

#### Other variables

Information on sociodemographic characteristics, were collected for both snakebite cases and non-cases, including sex, age and district of residence. Among snakebite cases, further sociodemographic, snakebite experience, healthcare information and other behavioral variables were also collected: sociodemographic characteristics (socioeconomic status, education), snakebite experiences (time of the snakebite, time of day, activities during the snakebite, geographical location of the bite, symptoms experienced), healthcare experiences (initial first aid measures taken), and other behavioral variables (precautions taken to protect against snakes, such as using mosquito nets, and whether the house was illuminated at night).

### Data sources and measurement

#### Data collection tools

This study employed two questionnaires for data collection: the household questionnaire and the care-seeking behavior questionnaire. The household questionnaire aimed to screen all individuals residing in the selected sectors for snakebite incidents at the household level. It collected information on participant consent, age, sex, socioeconomic status (referred to locally as ubudehe), whether the individual self-reported being a SBE victim in 2020 and contact information.

The care-seeking behaviors questionnaire was designed to assess care-seeking behaviors among human SBE victims and consisted of six sections: (1) geographic location, (2) demographics, (3) snakebite incidents, (4) medical signs/symptoms, (5) care-seeking decisions, and (6) SBE prevention practices. Both questionnaires were initially developed in English and then translated into Kinyarwanda. The care-seeking behaviors questionnaire was uploaded to Qualtrics and underwent pre-testing before implementation. All the study tools are available in S5 Appendix.

#### Data collection procedures

Data collection occurred during the SARS-Cov2 pandemic. Thus, protocols to train enumerators and collect data were adapted to minimize travel and avoid person-to-person contact. In March 2021, our team used a virtual platform to train hospital managers in charge of CHWs to conduct door-to-door surveys in Eastern Province, Rwanda. In turn, the managers trained CHWs from selected villages to complete our household survey on paper forms. CHWs were instructed to record everyone who was living in their village in 2020 and inquire if a snake had bitten the person and their contact information in addition to demographic characteristics. The exclusion criterion was not living in the village during 2020. CHWs were instructed to make three attempts on different days in case they didn’t find household members initially. The completed forms were returned to a central location for quality control. When forms were incomplete or contained errors, the CHW was requested to repeat data collection.

Completed household surveys were manually entered into an Excel spreadsheet and checked for errors. Individuals recorded as a human snakebite case on the household survey were called by telephone, asked to verify that they were bitten by a snake in 2020, and if confirmed as a true case, were invited to participate in the care-seeking survey. At least three attempts were made to contact each victim over seven days. If the victim had no telephone or their contact information was incorrect, our team recruited the village CHW to lend their telephone for the purpose of the interview. If the victim was a child, the interview was conducted with the parent or guardian; some victims near the age of majority chose to sit with the respondent during the interview. An interview log was maintained to document non-cases, interview refusals, and contact attempts. Those victims who consented to the telephone survey were interviewed by a Kinyarwanda-speaking enumerator who entered responses directly into the survey platform. Throughout data collection, Qualtrics backend data was monitored daily to ensure data quality. Any errors were immediately followed up and resolved.

#### Bias

To reduce bias, the study team implemented several quality control mechanisms throughout the study. These mechanisms included creating standardized study protocols and data entry sheets as well as organizing training sessions for CHWs (household survey) and enumerators (care-seeking survey) to ensure consistent data collection techniques. Backend data for the victim survey was monitored in real time to detect and correct inter-operator discrepancies. Study sectors were randomly selected, and all snakebite victims residing within those target areas during the relevant time frame were invited to participate in our study. A detailed description of the quality control mechanisms procedures, along with the study tools, is provided in S5 Appendix.

#### Study size

To determine the sample size, we utilized a regional estimate of SBE incidence in our sampling calculations, as there were no local estimates for SBE incidence available. The regional estimate included was 4,171 out of a population of 12,089,720 [5]. Additionally, we used a critical value of 1.96 for a 95% confidence interval, a margin of error of 0.00005, a Design Effect of 1.5 to account for cluster sampling and assumed a participation rate of 90%. This sample size required to be surveyed was 704,175 individuals.

We employed cluster sampling to select participants for the study, considering districts as strata and sectors as clusters. Table A in S1 Appendix illustrates the selection process. Using the latest government census data for 2020 [17], we estimated district populations (column 2) and the average population per sector in each district (column 4). Next, we calculated population proportions for each district (column 5), which were then applied to determine the number of individuals to select per district (column 6).

**Fig 1:**
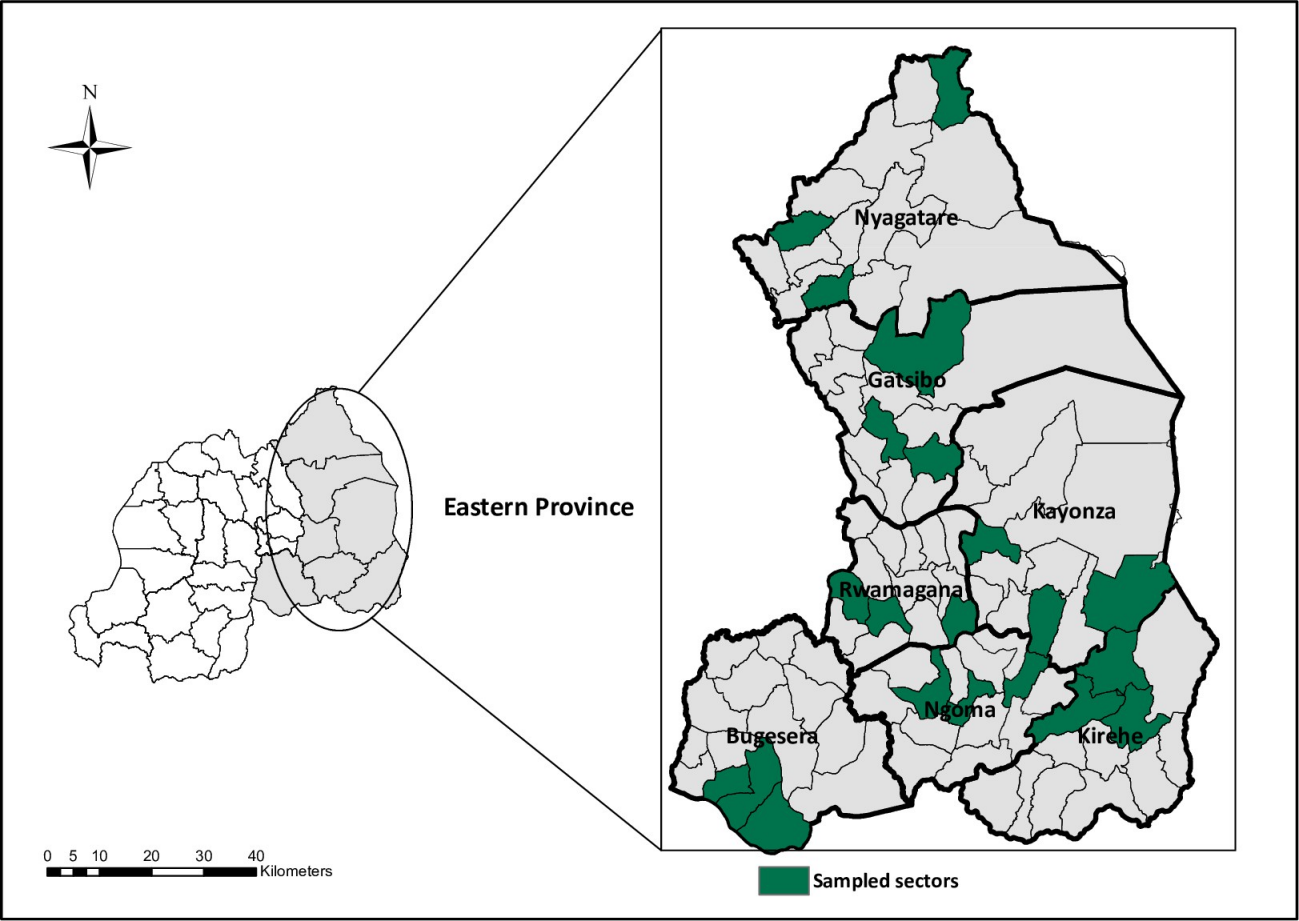
Map displaying selected sectors in Eastern Province (3 sectors per district). *The map was created by the author (DH) utilizing ArcMap 18*.*8*.*2*. *Its base layer comprises shapefiles sourced from the National Institute of Statistics of Rwanda and also publicly accessible via The World Bank (License*: *Creative Commons Attribution 4*.*0)*: *https*:*//datacatalog*.*worldbank*.*org/search/dataset/0041453/Rwanda-Admin-Boundaries-and-Villages*.

To reach the required sample size, we calculated the number of sectors (clusters) to select in each district based on the average population per sector (S1 Appendix). We rounded sectors up if the decimal was 5 or above. These calculations revealed that three clusters (sectors) should be randomly selected within each of the seven districts to achieve the required sample size. As a result, we randomly selected three sectors from each district, leading to the inclusion of 21 sectors, encompassing 814 villages in the study (Fig 1).

#### Statistical methods

Both the snakebite incidence survey and healthcare-seeking behaviors survey datasets were initially stored in Microsoft Excel and subsequently imported into STATA version 14.2 for data cleaning. The cleaned datasets were then analyzed using R version 4.3.0.

For all survey respondents, we conducted a descriptive statistical analysis and presented the frequencies and percentages of total confirmed survey cases, unconfirmed snakebite cases, and non-cases categorized by sex, age, and district of residence.

To estimate the snakebite incidence, we considered the cluster sampling design and accounted for the recorded cases that could not be reached by telephone to be confirmed. To address unconfirmed cases, we assumed they occurred randomly after further exploration revealed that their characteristics did not differ substantially from the reached cases. We then employed the random hot deck imputation method to estimate the probable number of unconfirmed cases, matching them by sector. First, sector-level proportions of confirmed cases to reached cases were calculated. Next, for observations with missing confirmation status, a random number was assigned to each observation within sectors where confirmation status was missing. Observations were then ordered based on these random numbers, and a unique ID was assigned to each observation. The dataset was joined with sector proportion information to determine the number of cases to be imputed for each sector. Observations to be imputed were identified by comparing the assigned ID with the number of cases not reached within each sector. Finally, missing confirmation statuses were imputed by replacing them with "Confirmed Case" for selected observations and "non-case" for any remaining missing values. This ensured that the same proportion of unreached cases within each sector was imputed. The detailed Imputation process is available in S2 Appendix.

In consideration of the cluster sampling design, we integrated sampling weights into our estimation. To calculate these weights, we initially determined the selection probability of each chosen participant within each district (considered as strata in our sampling) by dividing the sampled participants by the expected number within each district. Subsequently, we calculated the inverse of the selection probability for each sector. These weights were then applied to the dataset that included imputed cases to compute snakebite incidence and the number of snakebite cases per district and at the provincial level, using the survey package in R. This package utilizes the Horvitz-Thompson estimator and Taylor series linearization for variance estimation [18].

Data analysis of snakebite experience, healthcare seeking behaviors and other information on snakebite cases employed descriptive statistics and summarized the data using frequency and percentage distributions to present variations among snakebites victims.

## Results

### Snakebite incidence household survey results

#### Characteristics of study participants

**Fig 2:**
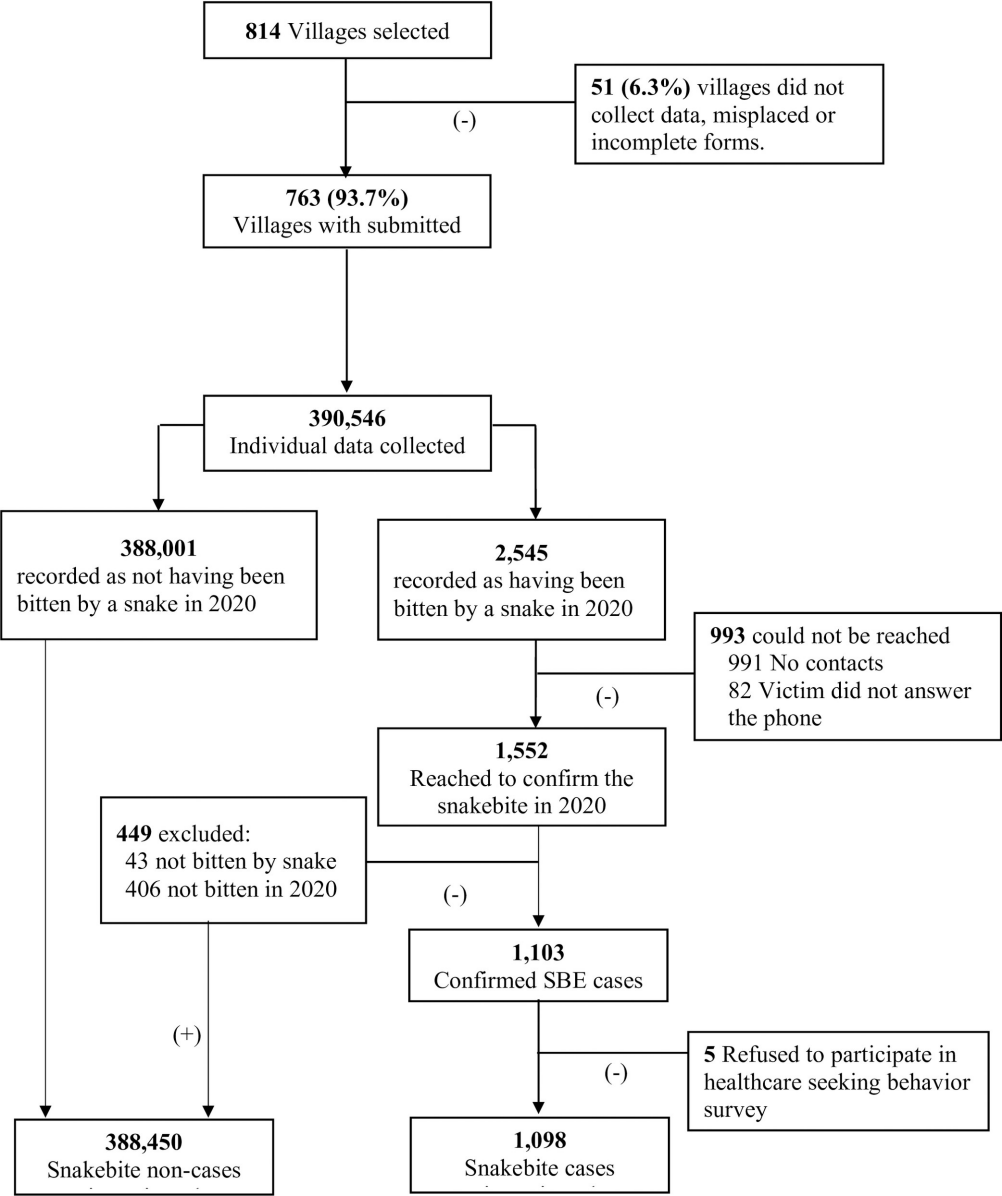
Data collection flow chart.

Of the 814 villages selected for data collection, we obtained complete information for 763 villages (93.7%; Fig 2). The other 51 villages were excluded because data were not collected, incomplete, or misplaced. Within the remaining 763 villages, we collected demographic data for 390,546 individuals, averaging 18,597 individuals per sector (Table 1). Additionally, 2,545 people were recorded by CHWs as having been bitten by snakes in 2020 (Fig 2). After multiple attempts to contact all recorded victims, our team successfully reached out to 1,552 individuals to ascertain their snakebite status. Of those reached, 1,103 individuals confirmed being bitten by a snake in 2020, while 449 reported no snakebite or no snakebite in 2020. These 449 individuals were classified as non-cases, contributing to a total of 388,450 non-cases (Table 2 and Fig 2).

**Table 1:** Data per sector (cluster) recorded from the household survey conducted in Eastern Province, Rwanda.

District (Stratum)	# Sectors (Clusters)	#Observations per sector			
		Total	Minimum	Mean	Maximum
Bugesera	3	70,233	18,972	23,411	32,125
Gatsibo	3	56,602	11,416	18,867	24,283
Kayonza	3	54,446	8,232	18,149	28,683
Kirehe	3	57,801	12,774	19,267	24,964
Ngoma	3	53,016	13,103	17,672	25,162
Nyagatare	3	53,527	11,997	17,842	26,800
Rwamagana	3	44,921	11,487	14,974	17,991
**Total**	**21**	**390,546**	**8,232**	**18,597**	**32,125**

**Table 2:** Demographic characteristics and 2020 snakebite status of individuals recorded from household survey conducted in Eastern Province, Rwanda (N = 390,546).

Variables and categories	Alive and reached via telephone/ Confirmed (N = 1,103)	Not reached via telephone/ Unconfirmed (N = 993)		Non-snakebite case (n %) (N = 388,450)	Total (n %) (N = 390,546)
		Alive (N = 990)	Deaths (N = 3)		
**District**					
Bugesera	366 (33.2)	316 (31.9)	-	69,551 (17.9)	70,233 (18.0)
Gatsibo	43 (3.9)	87 (8.8)	-	56,471 (14.5)	56,601 (14.5)
Kayonza	61 (5.5)	29 (2.9)	1 (33.3)	54,355 (14.0)	54,446 (13.9)
Kirehe	102 (9.2)	127 (12.8)	-	57,572 (14.8)	57,801 (14.8)
Ngoma	294 (26.7)	283 (28.6)	2 (66.7)	52,437 (13.5)	53,016 (13.6)
Nyagatare	49 (4.4)	13 (1.3)	-	53,465 (13.8)	53,527 (13.7)
Rwamagana	188 (17.0)	135 (13.6)	-	44,599 (11.5)	44,922 (11.5)
**Sex of the participant**					
Female	678 (61.5)	394 (39.8)	2 (66.7)	200756 (51.7)	20,1830 (51.7)
Male	424 (38.4)	277 (28.0)	1 (33.3)	183,674 (47.3)	18,4376 (47.2)
Not recorded	1 (0.1)	319 (32.2)	-	4,020 (1.0)	4,340 (1.1)
**Age categories**					
10–19	245 (22.2)	117 (11.8)	-	98,263 (25.3)	98,625 (25.3)
20–29	190 (17.2)	152 (15.4)	-	59,691 (15.4)	60,033 (15.4)
30–39	215 (19.5)	133 (13.4)	-	51,276 (13.2)	51,624 (13.2)
40–49	167 (15.1)	86 (8.7)	1 (33.3)	36419 (9.4)	36,673 (9.4)
50 and above	185 (16.8)	141 (14.2)	2 (66.7)	47,326 (12.2)	47,654 (12.2)
<10 years	93 (8.4)	41 (4.1)	-	92,018 (23.7)	92,152 (23.6)
Not recorded	8 (0.7)	320 (32.3)	-	3,457 (0.9)	3,785 (1.0)

#### Estimated SBE incidence

Table 3 presents the estimated snakebite cases and incidence per 1,000 population in Eastern Province districts. Our calculations indicate that the estimated incidence in 2020 for Eastern Province was 4.3 (95% CI: 3.0–6.2) snakebite cases per 1,000 population annually, totaling 13,509 cases (95% CI: 8329–18690). The highest estimated incidences were observed in Bugesera District (9.1, 95% CI: 5.4–15.5) and Ngoma District (9.1, 95% CI: 4.5–18.2), followed by Rwamagana District with an estimated incidence of 6.5 (95% CI: 3.5–12.2) cases per 1,000 population. In contrast, Nyagatare District had the lowest estimated incidence at 1.1 (95% CI: 0.6–1.9) per 1,000 population.

**Table 3:** Estimated snakebite cases and incidence per 1,000 population in Eastern Province districts, Rwanda, in 2020.

District	Incidence per 1,000 (95% CI)		Totals (95% CI)	
Bugesera	9.1	(5.4–15.5)	3,984	(579–7,389)
Gatsibo	1.6	(0.8–3.2)	838	(68–1,608)
Kayonza	1.6	(0.4–6.4)	670	(75–1,265)
Kirehe	3.1	(1.4–6.8)	1,276	(0–2,719)
Ngoma	9.1	(4.5–18.2)	3,673	(323–7,024)
Nyagatare	1.1	(0.6–1.9)	597	(428–767)
Rwamagana	6.5	(3.5–12.2)	2,470	(1,489–3,452)
**Province**	**4.3**	**(3.0–6.2)**	**13,509**	**(8,329–18,690)**

Coefficients of variation: for non-cases: 0.07; for snakebite cases: 16.6

Design Effect for variance: 53.6

### Healthcare-seeking behaviors

#### Characteristics of study participants

A total of 1,098 snakebite victims from the seven districts in Eastern Province participated in our healthcare-seeking survey (Table 4). The majority were female (61.6%), aged between 10 and 39 years (59.1%), had low ubudehe status (68.4%), and resided in Bugesera (33.2%) or Ngoma districts (26.6%). Furthermore, most respondents had completed primary education (58.8%). Most respondents identified as farmers (64.1%), while a smaller percentage were either unemployed or children (16.7%). A significant portion had health insurance coverage (89.2%).

**Table 4:** Demographic characteristics versus first point of care of individuals bitten by snakes in Eastern Province, Rwanda in 2020.

Variables	Categories	Formal care^1^ (n, %)	Informal care (n, %)	Total (n, %) (N = 1,098)
Overall		143 (13%)	955 (87%)	
District	Bugesera	21 (14.7)	344 (36.0)	365 (33.2)
	Gatsibo	6 (4.2)	36 (3.8)	42 (3.8)
	Kayonza	16 (11.2)	45 (4.7)	61 (5.6)
	Kirehe	20 (14.0)	82 (8.6)	102 (9.3)
	Ngoma	48 (33.6)	243 (25.4)	291 (26.5)
	Nyagatare	6 (4.2)	43 (4.5)	49 (4.5)
	Rwamagana	26 (18.2)	162 (17.0)	188 (17.1)
Age categories	< 10 years	17 (11.9)	76 (8.0)	93 (8.5)
	10–19	29 (20.3)	215 (22.5)	244 (22.2)
	20–29	21 (14.7)	169 (17.7)	190 (17.3)
	30–39	31 (21.7)	186 (19.5)	217 (19.8)
	40–49	25 (17.5)	141 (14.8)	166 (15.1)
	50 and above	20 (14.0)	164 (17.2)	184 (16.8)
	Missing	0 (0)	4 (0.4)	4 (0.4)
Sex of the victim	Female	94 (65.7)	582 (60.9)	676 (61.6)
	Male	49 (34.3)	373 (39.1)	422 (38.4)
Highest education	No education	42 (29.4)	325 (34.0)	367 (33.4)
	Primary	88 (61.5)	558 (58.4)	646 (58.8)
	Secondary or Higher	13 (9.1)	71 (7.4)	84 (7.7)
	Missing	0 (0)	1 (0.1)	1 (0.1)
Social economic level (Ubudehe)	1 (poorest)	24 (16.8)	163 (17.1)	187 (17)
	2	70 (49.0)	493 (51.6)	563 (51.3)
	3 & 4 (wealthiest)	49 (34.3)	299 (31.3)	348 (31.7)
Primary occupation	Unemployed	14 (9.8)	138 (14.5)	152 (13.8)
	Casual labor	11 (7.7)	33 (3.5)	44 (4)
	Farmer	84 (58.7)	593 (62.1)	677 (61.7)
	Professional works	7 (4.9)	34 (3.6)	41 (3.7)
	Student/Child	27 (18.9)	157 (16.4)	184 (16.8)
Household size	1–3	25 (17.5)	121 (12.7)	146 (13.3)
	4–6	77 (53.8)	385 (40.3)	462 (42.1)
	Above 6	20 (14.0)	105 (11.0)	125 (11.4)
	Not recorded	21 (14.7)	344 (36.0)	365 (33.2)
Had health insurance in 2020	No	13 (9.1)	106 (11.1)	119 (10.8)
	Yes	130 (90.9)	849 (88.9)	979 (89.2)

^1^The first point of care following the snakebite; formal is defined as seeking care from a Community Health Worker, health post/center or hospital whereas informal is defined as family/friends, pharmacist, or traditional healer

#### Healthcare-seeking behaviors and experiences

**Table 5** shows that a significant proportion of snakebites occurred during nighttime (66.3%), and about half of the respondents perceived the bite as potentially life-threatening (53.3%). In the majority of cases, respondents identified their snakebite by witnessing the attack (65.4%) or observing clear fang marks (29.0%). A notable number of respondents (40.7%) reported killing the snake. Notably, only 143 victims (13%) sought formal care (CHWs, health post/center or hospital) as their initial point of contact following the snakebite, while the remaining 955 individuals (87%) consulted informal care (family/friends, pharmacist, or traditional healer). When considering the perceived providers offering the highest quality snakebite care, traditional healers (46.4%) and health posts/health centers (41.1%) were identified as the top choices (**Table 5**).

**Table 5:** Description of snakebite experiences in Eastern Province, Rwanda in 2020.

Variables	Categories	Formal care^1^ (N = 143)	Informal care (N = 955)	Total (N = 1,098)
		n (%)		
# snakebites in 2020	One time	136 (95.1)	917 (96.0)	1,053 (95.9)
	>1	7 (4.9)	38 (4.0)	45 (4.1)
Time of day when bite occurred	Day	44 (30.8)	321 (33.6)	365 (33.2)
	Night	99 (69.2)	629 (65.9)	728 (66.3)
	Not specified	0 (0)	5 (0.5)	5 (0.5)
How knew was bitten	Fang marks visible/felt bite	40 (28.0)	288 (30.2)	328 (29.9)
	Saw the snake bite or spit	94 (65.7)	624 (65.3)	718 (65.4)
	Others	9 (6.3)	43 (4.5)	52 (4.7)
Anyone identified the snake	No	46 (32.2)	265 (27.7)	311 (28.3)
	Yes	97 (67.8)	690 (72.3)	787 (71.7)
Killed the snake	No	89 (62.2)	562 (58.8)	651 (59.3)
	Yes	54 (37.8)	393 (41.2)	447 (40.7)
Self-reported severity level	Mild to moderate	20 (14.0)	128 (13.4)	148 (13.5)
	Very serious	102 (71.3)	481 (50.4)	583 (53.1)
	Not reported	21 (14.7)	346 (36.2)	367 (33.4)
Perceived provider offering the highest quality SBE care	Health post/center	99 (69.2)	352 (36.9)	451 (41.1)
	Hospital	27 (18.9)	58 (6.1)	85 (7.7)
	Traditional healer	11 (7.7)	498 (52.1)	509 (46.4)
	Undecided	6 (4.2)	40 (4.2)	46 (4.2)
	Community health worker	0 (0)	6 (0.6)	6 (0.5)
	Pharmacist	0 (0)	1 (0.1)	1 (0.1)

According to respondents, the most frequent symptoms were swelling (93.3%) and pain (87.7%), followed by blurred vision/dizziness (55.5%), numbness (54.2%), lethargy (51.4%), and gastrointestinal distress (49.8%;—see Table 6). Reports of uncontrolled bleeding (2.7%), blindness (11.4%), or paralysis (15.9%) were less common. Few respondents reported no symptoms. During the survey, we also inquired about whether the victims survived, and only three deaths were reported as related to snakebite. Among the 583 snakebite victims who reported severe symptoms, pain and inflammation were highly prevalent (96.2%), alongside lethargy (81.0%) and nausea/vomiting/diarrhea (80.1%). Difficulty breathing was noted in 57.8%, while blurred vision/dizziness and numbness were also common (64.0% and 64.3%, respectively). Paralysis, loss of consciousness, and blindness were less frequently reported (18.7% to 26.9%). Uncontrolled bleeding, skin rashes, and fever were infrequent, affecting less than 10% of individuals (S4 Appendix).

**Table 6:** Self-reported symptoms versus first point of care among snakebite victims in Eastern Province, Rwanda in 2020^1^.

Symptoms	Formal care (n, %)	Informal care (n, %)	Total (n, %)
	(N = 143)	(N = 955)	(N = 1,098)
Pain	131 (91.6)	830 (86.9)	961 (87.5)
Swelling/Inflammation	138 (96.5)	886 (92.8)	1,024 (93.3)
Uncontrolled bleeding	3 (2.1)	27 (2.8)	30 (2.7)
Difficulty breathing	72 (50.3)	327 (34.2)	399 (36.3)
Sweating	54 (37.8)	244 (25.5)	298 (27.1)
Lethargy	96 (67.1)	468 (49.0)	564 (51.4)
Nausea, vomiting or diarrhea	103 (72.0)	444 (46.5)	547 (49.8)
Blurred vision/dizziness	89 (62.2)	520 (54.5)	609 (55.5)
Numbness	87 (60.8)	508 (53.2)	595 (54.2)
Paralysis	35 (24.5)	140 (14.7)	175 (15.9)
Loss of consciousness	39 (27.3)	186 (19.5)	225 (20.5)
Blindness	20 (14.0)	105 (11.0)	125 (11.4)
Skin rashes and tongue	7 (4.9)	49 (5.1)	56 (5.1)
Fever	4 (2.8)	16 (1.7)	20 (1.8)
None	0 (0)	3 (0.3)	3 (0.3)

^1^Respondents could select more than one symptom

Table 7 shows that almost three-quarters (73.9%) of respondents reported receiving first aid immediately after the snakebite. This included tourniquet application (58.4%), burning the wound (20.9%), and ingesting or applying herbs (11.2%). Victims received care from traditional healers (84.0%), health centers (23.6%), hospitals (3.9%), pharmacists (1.2%), and CHWs (0.9%). The types of care received varied based on the provider. Traditional healers mainly administered pain relief/anti-swelling (38.1%) and herbal drinks/salves (34.2%); pharmacies primarily dispensed pain relief/anti-swelling (61.5%). Health centers dispensed pain relief/anti-swelling (68%), and hospitals provided pain relief/anti-swelling (72.1%), with 51.2% receiving anti-venom.

**Table 7:** Treatment provided to Rwandese snakebite victims from Eastern Province in 2020 by care providers, as reported by respondents^1^.

Care provided	Level of care (n, %)					
	First Aid	Traditional	Pharmacy	CHW^2^	HP/HC^2^	Hospital
	(n = 1,098)	(n = 922)	(n = 13)	(n = 10)	(n = 259)	(n = 43)
Tourniquet	641 (58.4)	125 (13.6)	-	1 (10.0)	2 (0.8)	-
Burning skin	230 (20.9)	112 (12.1)	-	-		-
Cutting skin	31 (2.8)	83 (9.0)	-	-	5 (1.9)	-
Sucking venom	24 (2.2)	106 (11.5)	1 (7.7)	-	22 (8.5)	6 (14.0)
Herbal drinks/salves	123 (11.2)	315 (34.2)		-		
Black stone	42 (3.8)	191 (20.7)	1 (7.7)	2 (20.0)	66 (25.5)	7 (16.3)
Pain relief/anti swelling	-	351 (38.1)	8 (61.5)	1 (10.0)	176 (68.0)	31 (72.1)
Anti-venom^4^	-	-	2 (15.4)	-	-	22 (51.2)
Other	-	381 (41.3)	4 (30.8)	4 (40.0)	92 (35.5)	25 (58.1)
Referral	-	6 (0.7)	-	4 (40.0)	15 (5.8)	-
Unsure	-	32 (3.5)	-	-	4 (1.5)	-
No treatment	287 (26.1)	5 (0.5)	1 (7.7)	1 (10.0)	6 (2.3)	1 (2.3)

^**1**^Victim could receive more than one care.

^**2**^CHW = Community Health Worker

^**3**^HPHC = Health post/health center

^**4**^Of the 24 cases that received anti-venom, 23 were identified as severe cases, while only one was categorized as moderate.

**Fig 3:**
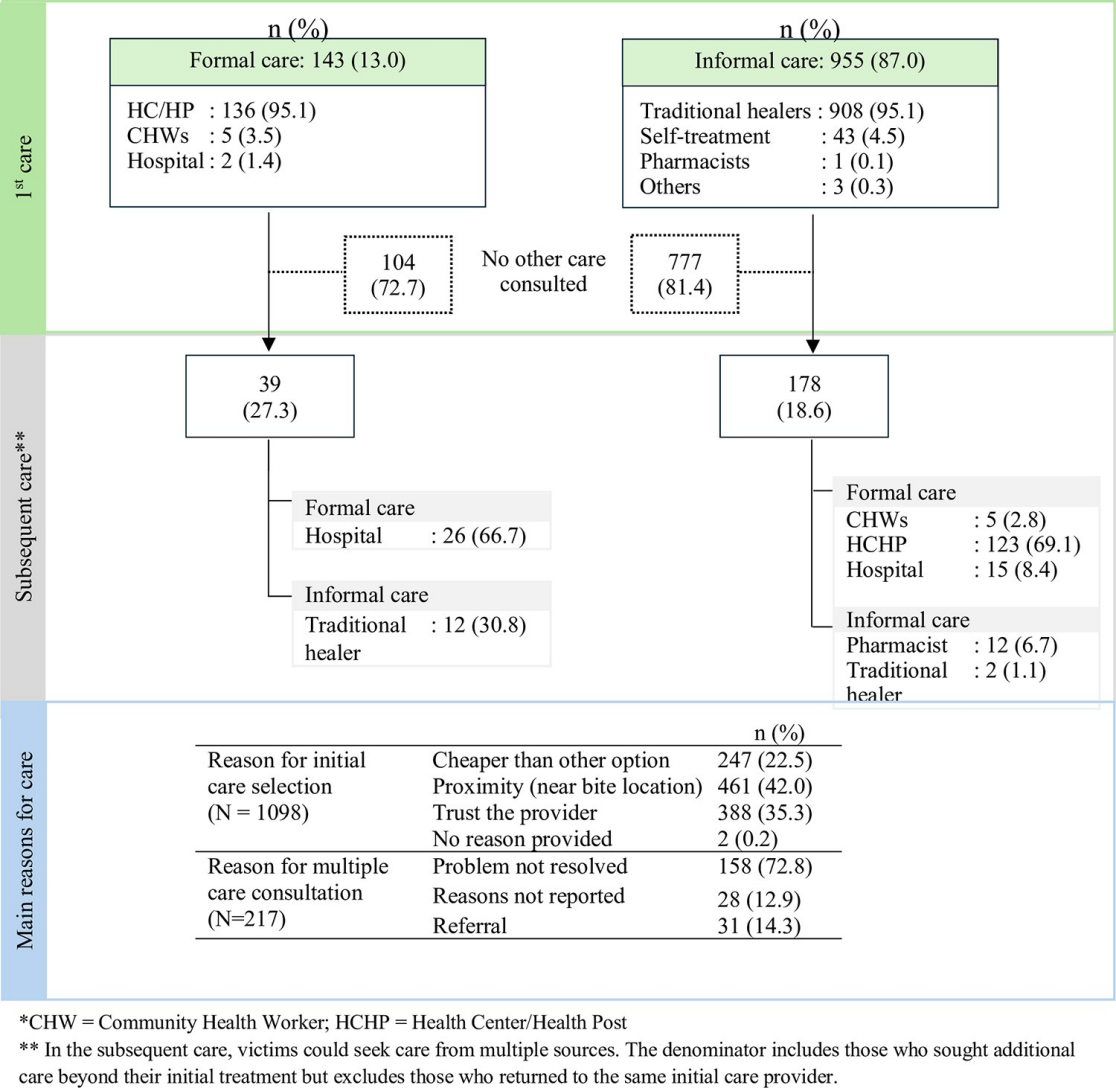
Movement of snakebite patients between formal and informal care systems and reasons for choosing care in Eastern Province, Rwanda in 2020.

Fig 3 illustrates crossover between healthcare systems, with 39 (27.3%) initially seeking formal care, and 178 (18.6%) of those initially consulting formal care providers subsequently seeking additional care. Out of the 39 individuals who initially consulted formal care, 30.8% also sought traditional healers. Among the 178 who initially opted for informal care, the majority (69.1%) visited health centers or health posts, while only 8.4% went to hospitals. Respondents cited proximity to the bite location (42.0%), trust in the provider’s ability to treat snake bites (35.3%), and cost-effectiveness (22.5%) as the main reasons for choosing their initial care. Among those seeking secondary care, 72.8% mentioned unresolved problems as the primary reason.

#### Snakebite prevention

Most participants believed their homes were susceptible to snake entry (85.3%) and lacked adequate nighttime lighting (65.6%; Table 8). About half (51.7%) implemented measures to control rodents, including traps, chemicals, cleaning, or owning a cat. Most (58.8%) reported always carrying a torch at night, and 73.6% indicated they always slept under a mosquito net.

**Table 8:** Preventive measures for rodents and snakebites.

Variables	Categories	Total (n %)
Carry a torch when walking at night (n = 1,094*)	Always	643 (58.8)
	Never	109 (10)
	Sometimes	342 (31.3)
Sleep under a mosquito net (n = 1,094)	Always	805 (73.6)
	Never	95 (8.7)
	Sometimes	194 (17.7)
Perceives house is secured from snakes entering (n = 1,087)	No	927 (85.3)
	Yes	160 (14.7)
Perceives house is well lighted at night (n = 1095)	No	718 (65.6)
	Yes	377 (34.4)
Has measures to control rodents (n = 1094)	No	528 (48.3)
	Yes	566 (51.7)
Measures to control rodents (among those with measures (N:566)	Traps	359 (63.4)
	Chemicals	225 (39.6)
	Kill them	95 (16.8)
	Cats	58 (10.3)
	Cleaning	41 (7.2)

*Some participants skipped questions, which is why the N varies.

## Discussion

### Key results and interpretation

This first door-to-door assessment of snakebite in Rwanda confirms the serious under-reporting of snakebite as well as the overwhelming use of traditional medicine. Our team estimated an annual snakebite incidence for one province alone (13,509 ; 95% CI: 8,329–18,690) that was more than two times higher than the latest national SBE estimate (4,171; 95% CI 3,454–4,885) obtained through meta-analysis [5]. It far exceeds the 61–72 cases reported by Eastern Province hospitals to the Health Information Management System in 2017–2018, and confirms the higher case counts among women versus men [4,6]. The World Health Organization estimates that up to 60% of snakebite cases could be ‘dry’ or non-venomous, suggesting that at least 5,404 individuals in Eastern Province experienced SBE and would have benefitted from expert medical care [12].

Despite half of our respondents’ (53.5%) belief that their bite was life-threatening, only two (0.2%) victims presented to hospitals as a first point of care. Most (90.6%) sought informal care. In Rwanda, public hospitals alone stock snake anti-venom [8], which is the only remedy with proven efficacy in neutralizing snake venom. Altogether, this paints a concerning picture for achieving the Ministry of Health 2024 goals for SBE reduction [13]. There is a clear need to develop community-based surveillance systems that report cases regardless of care provider, to strengthen programs for snakebite prevention, to engage the public in appropriate care-seeking behavior, and to improve overall case management.

Across SSA, the epidemiology and geographic distribution of SBE is not well characterized [19]. SBE victims who seek care from traditional healers are not recorded in government public health surveillance systems. This means that incidence calculations based on hospital records significantly under-estimate the true epidemiological and economic burden [20,21]. A community-based assessment in Mozambique, for example, resulted in a ten-fold higher estimate of SBE incidence than previous assessments [21]. Our annual incidence estimate for Eastern Province of 4.3 cases per 1,000 people falls within the SSA regional estimate for SBE of 1.0–6.5 cases per 1,000 and is similar to that reported by other household surveys in Cameroon (6.7 cases/1,000), Mozambique (3.5 cases/1,000), and rural Africa (2.0 cases/1,000) [20–23]. However, the district estimates for Bugasera and Ngoma (9.1 cases/1,000) are far higher. The scarcity of community-based estimates of SBE might help to explain the disconnect between SBE burden and resource mobilization in African public health systems and suggest a need to develop improved surveillance systems [20,21,24].

The strong preference for traditional medicine observed in this study was higher than that previously reported in Mozambique (59%), Kenya (30–53.8%) or Cameroon (38%) [21,22,25,26]. These variations are likely due to differences in informal care definitions but could also reflect perceptions of physician competence in SBE management. In Rwanda, traditional healers are popular because they promise to treat both the medical and spiritual components of snakebite, also offering protection against future bites [9]. Many traditional healers make home visits and offer flexible repayment plans that include labor and goods in exchange for services, making such services appear both affordable and convenient [9]. Unfortunately, traditional medicine has no proven efficacy against SBE; worse yet, it causes delays to formal health seeking that worsen patient outcomes [27,28]. Popular practices such as wound burning, cutting and tourniquet can cause additional damage and increase the risk of long-term disability [28]. Unfortunately, snakebite victims often hesitate to seek formal medical services due to the perceptions that physicians are not trained to treat snakebite. This poor public perception of SBE services offered by Rwandan hospitals is supported by recent data demonstrating low physician confidence, inadequate training, and frequent stock-outs of essential commodities, such as snake anti-venom [7,8]. Hospital care did not meet the World Health Organization threshold for affordability, with a single anti-venom dose costing more than 10 days of wages from a low income worker with no health insurance [8]. These issues are not unique to Rwanda but justify investments to improving formal medical services and engaging the public on appropriate snakebite treatment. The mismatch between respondent perspectives on best care providers (49.3% in favor of formal care) compared to their practices suggests that victims are willing to adapt, given appropriate support.

Preventing snakebite is a key component of burden reduction, especially in resource poor settings where morbidity management can face numerous challenges [29]. Measures to avoid snakebite can include wearing shoes, carrying a torch at night, sleeping off the ground under a well-tucked mosquito net, storing food appropriately, and controlling rodent populations [29]. Snakebite victims in this study lived predominantly in homes with poor lighting and with infrastructure inadequate for preventing snake entry. Only half of respondents employed measures to control rodents. These findings align with previous observations demonstrating poor knowledge and practices of snakebite prevention and unsupported beliefs that killing snakes reduces injuries [7,9]. They also align with international recommendations, such as those developed during the 2015 Hinxton Retreat, to encourage community prevention campaigns and to improve first aid knowledge [30,31]. Our study did not examine why Rwandese respondents sharing environments with venomous snakes did not take more care to avoid unwanted contact, but poverty and poor knowledge are likely culprits. Elsewhere, snakebite education campaigns have demonstrated improved prevention practices, highlighting the role for community educators in supporting primary prevention [29].

### Strengths and limitations

This study had several limitations. Of the 2,545 individuals who self-reported snakebite in 2020, we were only able to verify bite status for 61% of potential cases. This is likely explained by the transient nature and telephone ownership status of typical victims in rural Rwanda. To minimize this issue, we employed CHWs to find recorded cases and used hot deck imputation to predict the real snakebite status of unconfirmed cases. We did not consider that recorded non-cases might actually be cases as snakebite is a traumatic event that people are generally willing to report. All data related to snakebite severity, treatment methods, and recovery were self-reported, and we were unable to determine whether individuals had been envenomed. Verbal autopsy was not performed. While patients often stated that they underwent a complete recovery following care, they later characterized ongoing physical and psychological symptoms. Therefore, it is likely that typical Rwandese patients equate survival with recovery. Our enumerators employed probes throughout data collection to better understand snakebite victims’ experiences. Finally, this study did not collect sufficient data at the village level to understand differences in district level snakebite risk. Additional information is needed to understand why two non-adjacent districts, Bugasera and Ngoma, appear to experience the highest number of cases. This study also had several strengths. First and foremost, snakebite data was collected in villages, rather than at hospitals or clinics, ensuring that all snakebite victims could be enumerated regardless of care seeking behavior. Second, snakebite data was collected over a broad geographic area with a large sample, allowing our team to capture district level nuances in risk and decision making. Third, our methodology demanded strong quality control at all stages to ensure that data were entered accurately, cases were verified, and uncertainties were accounted for in the statistical analysis.

## Conclusion

This work demonstrates a significant burden of snakebites in Rwanda, with incidence rates varying widely across districts. Despite the severity of the condition, a substantial portion of victims initially sought informal care, highlighting the need for increased awareness and access to formal healthcare services. Unsafe practices such as skin cutting/burning and tourniquet application were common, underscoring community education’s importance in promoting safer management strategies. Anti-venom administration was infrequent, indicating a critical gap in treatment availability and delivery. Addressing these challenges requires collaborative efforts between healthcare providers, policymakers, traditional healers, and community stakeholders to improve awareness, access to care, and the implementation of effective snakebite management strategies. Additionally, more attention is needed to support vulnerable communities in preventing human-snake conflicts.

## Supporting information

S1 Appendix Eastern Province population estimates. (DOC)

S2 Appendix Imputation Method Protocol. (DOC)

S3 Appendix Consent and Information Form (English version). (DOC)

S4 Appendix Symptoms among the self-reported severe cases. (DOC)

S5 Appendix Quality Control Mechanisms and Study Tools. (DOC)
