# Supplementary material for: Snakebite incidence and healthcare-seeking behaviors in Eastern Province, Rwanda: A cross-sectional study
Source: PLoS Negl Trop Dis. 2024 Aug 21;18(8):e0012378. doi: 10.1371/journal.pntd.0012378 (PMC11338457; doi:10.1371/journal.pntd.0012378)
Supplement: S1 Appendix — (DOC) [file pntd.0012378.s001.doc]

**Snakebite incidence and healthcare-seeking behavior in Eastern Province, Rwanda: A cross-sectional study**

Dieudonne Hakizimana^1,2^*, Lauren E. MacDonald^3^, Happy Tahirih Kampire^4^; Mihigo Bonaventure^4^; Mahlet Tadesse^4^, Elijah Murara^4^; Leila Dusabe^4^, Leandre Ishema^4^, Janna M. Schurer^4,5^*

**Table A. Eastern Province population estimates**

| **District** | **2020 Population** | **# Sectors** | **Mean Population/ Sector** | **Population Proportion/ District** | **Sample population/ District** | **# Sectors (clusters)/District** |
| --- | --- | --- | --- | --- | --- | --- |
| Rwamagana | 377,463 | 14 | 26,962 | 12.1% | 85,037 | 3.2 (3) |
| Nyagatare | 560,973 | 14 | 40,070 | 17.9% | 126,379 | 3.2 (3) |
| Gatsibo | 521,434 | 14 | 37,245 | 16.7% | 117,472 | 3.2 (3) |
| Kayonza | 414,427 | 12 | 34,536 | 13.3% | 93,365 | 2.7 (3) |
| Kirehe | 409,864 | 12 | 34,155 | 13.1% | 92,337 | 2.7 (3) |
| Ngoma | 405,722 | 14 | 28,980 | 13.0% | 91,404 | 3.2 (3) |
| Bugesera | 435,809 | 15 | 29,054 | 13.9% | 98,182 | 3.4 (3) |
| **Total** | **3,125,692** | **95** | **32,902** | **1** | **704,175** | **21** |
