# Supplementary material for: Snakebite incidence and healthcare-seeking behaviors in Eastern Province, Rwanda: A cross-sectional study
Source: PLoS Negl Trop Dis. 2024 Aug 21;18(8):e0012378. doi: 10.1371/journal.pntd.0012378 (PMC11338457; doi:10.1371/journal.pntd.0012378)
Supplement: S2 Appendix — (DOC) [file pntd.0012378.s002.doc]

**Snakebite incidence and healthcare-seeking behavior in Eastern Province, Rwanda: A cross-sectional study**

Dieudonne Hakizimana^1,2^*, Lauren E. MacDonald^3^, Happy Tahirih Kampire^4^; Mihigo Bonaventure^4^; Mahlet Tadesse^4^, Elijah Murara^4^; Leila Dusabe^4^, Leandre Ishema^4^, Janna M. Schurer^4,5^*

**S2 Appendix**

**Imputation Method Protocol:**

This protocol outlines the step-by-step procedure for imputing missing confirmation statuses using a random hot-deck method based on sector-level proportions and random number generation within sectors.

**Step 1: Sector Proportion Calculation:**

- Group the dataset by sector (cluster).
- Calculate the total number of recorded cases, reached cases, and confirmed cases within each sector.
- Compute the proportion of confirmed cases to reached cases for each sector.
- Determine the number of cases not reached within each sector.

**Step 2: Random Number Generation:**

- Assign a random number to each observation within sectors where confirmation status is missing.
- Ensure each observation receives a unique random number.
- This step is crucial for subsequent random selection of cases for imputation.

**Step 3: Random Selection of Cases for Imputation:**

- For observations with missing confirmation status, prioritize cases based on the random number generated in the previous step.
- Use the generated random numbers to order the observations within each sector.
- Assign a unique ID to each observation based on the ordered sequence.
- Join the dataset with the sector proportion information to determine the number of cases to be imputed for each sector.
- Identify the observations to be imputed by comparing the assigned ID with the number of cases not reached within each sector.

**Step 4: Imputation:**

- Replace the missing confirmation status with "Confirmed Case" for observations identified for imputation.
- Ensure that non-missing confirmation statuses remain unchanged.
- Replace any remaining missing confirmation statuses with "Non-case" to complete the imputation process.

**Table A:** Proportion of cases reached and imputed per sector

| Sector | Recorded cases | Reached cases | Confirmed cases | Proportion confirmed/reached | Total not reached | Imputed cases not reached |
| --- | --- | --- | --- | --- | --- | --- |
| Gashanda | 232 | 150 | 121 | 0.80666667 | 82 | 66 |
| Kabarore | 61 | 38 | 5 | 0.13157895 | 23 | 3 |
| Kamabuye | 71 | 71 | 71 | 1 | 0 | 0 |
| Karama | 23 | 20 | 18 | 0.9 | 3 | 3 |
| Kibungo | 88 | 65 | 23 | 0.35384615 | 23 | 8 |
| Kiziguro | 40 | 15 | 11 | 0.73333333 | 25 | 18 |
| Matimba | 54 | 47 | 19 | 0.40425532 | 7 | 3 |
| Mimuri | 25 | 22 | 12 | 0.54545455 | 3 | 2 |
| Mukarange | 28 | 23 | 23 | 1 | 5 | 5 |
| Munyaga | 149 | 93 | 83 | 0.89247312 | 56 | 50 |
| Murama | 9 | 9 | 9 | 1 | 0 | 0 |
| Mushikiri | 143 | 72 | 61 | 0.84722222 | 71 | 60 |
| Muyumbu | 126 | 75 | 55 | 0.73333333 | 51 | 37 |
| Nasho | 119 | 82 | 30 | 0.36585366 | 37 | 14 |
| Ndego | 58 | 33 | 29 | 0.87878788 | 25 | 22 |
| Ngeruka | 409 | 207 | 185 | 0.89371981 | 202 | 181 |
| Nyarubuye | 70 | 51 | 11 | 0.21568627 | 19 | 4 |
| Nzige | 95 | 68 | 50 | 0.73529412 | 27 | 20 |
| Remera | 79 | 40 | 27 | 0.675 | 39 | 26 |
| Ruhuha | 246 | 132 | 110 | 0.83333333 | 114 | 95 |
| Zaza | 420 | 240 | 150 | 0.625 | 180 | 112 |
| Total | 2545 | 1553 | 1103 | 0.71023825 | 992 | 729 |
