# Supplementary material for: Snakebite incidence and healthcare-seeking behaviors in Eastern Province, Rwanda: A cross-sectional study
Source: PLoS Negl Trop Dis. 2024 Aug 21;18(8):e0012378. doi: 10.1371/journal.pntd.0012378 (PMC11338457; doi:10.1371/journal.pntd.0012378)
Supplement: S3 Appendix — (DOC) [file pntd.0012378.s003.doc]

**Snakebite incidence and healthcare-seeking behavior in Eastern Province, Rwanda: A cross-sectional study**

Dieudonne Hakizimana^1,2^*, Lauren E. MacDonald^3^, Happy Tahirih Kampire^4^; Mihigo Bonaventure^4^; Mahlet Tadesse^4^, Elijah Murara^4^; Leila Dusabe^4^, Leandre Ishema^4^, Janna M. Schurer^4,5^*

**S3 Appendix: Consent and Information Form (English version)**

**Snakebite envenomation in Rwanda: A One Health assessment of incidence, demographics, and care-seeking behaviors**

**CONSENT AND INFORMATION FORM**

**Principal Researcher**:

Dr. Janna Schurer, Assistant Professor

Contact: [jschurer@ughe.org](mailto:jschurer@ughe.org)

**Co-Investigator:**

Dieudonne Hakizimana, Lecturer

Contact: [dhakizimana@ughe.org](mailto:dhakizimana@ughe.org)

Dear Participant,

You are being invited to participate in a research project on snakebites that is led by the University of Global Health Equity in Butaro. Before starting, we would like to provide you with important information that will help you to decide whether you want to participate.

The project has received required ethical approval from UGHE and complies with international ethical standards for research.

**The purpose of this project**

The purpose of this project is to estimate the number of people bitten by snakes each year and the ways they obtain medical treatment. We are also seeking information on domestic animals bitten by snakes.

**The procedure for participation in this project**

If you chose to participate, then we will conduct the survey by phone. We anticipate that this survey will take approximately 10 minutes to complete the questions.

**The possible benefits of taking part in this project**

Your participation will help us understand the magnitude of snakebites as a medical and veterinary problem in Rwanda and it will allow us to advise the government on how to improve programs to prevent snakebite and improve treatment.

**Possible risks or discomforts related to taking part in this project**

We anticipate that it might be difficult to talk your experience with snakebite or about a family member who died of snakebite. You are free to skip any question or to stop the survey at any time.

**Protection of your privacy**

The information collected will be kept confidential. Your personal information and responses will not be shared with anyone outside the research team. All project data will be kept safe in a password protected computer and destroyed after 10 years.

**Participation is voluntary**

It is your right to decide to participate in this project or not. If you choose to participate, you may change your mind and leave the study at any time. Refusal to participate or stopping your participation will involve no penalty.

**If I have any questions, concerns or complaints about this project, who can I talk to?**

In the event that you have questions about this research, please contact Dieudonne Hakizimana by phone (0788316894) or email ([dhakizimana@ughe.org](mailto:dhakizimana@ughe.org)).

In case you have concerns or complaints about this project, please contact the UGHE-IRB by email: [irb@ughe.org](mailto:irb@ughe.org)

**Statement of consent**

Your verbal consent indicates you acknowledge that:

1. You have understood the content of this form.
2. You have had the opportunity to ask questions and received answers that were satisfactory.
3. If needed, you took time to discuss this information with others to help you decide whether to participate.
4. You agree to participate in this project.

   Do you give verbal consent to participate in this survey?
   ◻ Yes

   ◻ No
