# Supplementary material for: Snakebite incidence and healthcare-seeking behaviors in Eastern Province, Rwanda: A cross-sectional study
Source: PLoS Negl Trop Dis. 2024 Aug 21;18(8):e0012378. doi: 10.1371/journal.pntd.0012378 (PMC11338457; doi:10.1371/journal.pntd.0012378)
Supplement: S4 Appendix — (DOC) [file pntd.0012378.s004.doc]

**Snakebite incidence and healthcare-seeking behavior in Eastern Province, Rwanda: A cross-sectional study**

Dieudonne Hakizimana^1,2^*, Lauren E. MacDonald^3^, Happy Tahirih Kampire^4^; Mihigo Bonaventure^4^; Mahlet Tadesse^4^, Elijah Murara^4^; Leila Dusabe^4^, Leandre Ishema^4^, Janna M. Schurer^4,5^*

**S4 Appendix**

**Table A:** Symptoms among the self-reported severe cases

| Categories | Formal care  (N=102) | Informal care(N=481) | Total  (N=583) |
| --- | --- | --- | --- |
| Pain | 97 (95.1) | 464 (96.5) | 561 (96.2) |
| Inflammation | 99 (97.1) | 462 (96.0) | 561 (96.2) |
| Uncontrolled bleeding at the bite site | 3 (2.9) | 21 (4.4) | 24 (4.1) |
| Difficulty breathing | 63 (61.8) | 274 (57.0) | 337 (57.8) |
| Sweating | 45 (44.1) | 207 (43.0) | 252 (43.2) |
| Lethargy | 83 (81.4) | 389 (80.9) | 472 (81.0) |
| Nausea, vomiting or Diarrhea | 90 (88.2) | 377 (78.4) | 467 (80.1) |
| Blurred vision/Dizziness | 67 (65.7) | 306 (63.6) | 373 (64.0) |
| Numbness | 70 (68.6) | 305 (63.4) | 375 (64.3) |
| Paralysis | 32 (31.4) | 125 (26.0) | 157 (26.9) |
| Loss of consciousness | 34 (33.3) | 171 (35.6) | 205 (35.2) |
| Blindness | 17 (16.7) | 92 (19.1) | 109 (18.7) |
| Skin rashes and tongue | 6 (5.9) | 37 (7.7) | 43 (7.4) |
| Fever | 4 (3.9) | 15 (3.1) | 19 (3.3) |
