# Supplementary material for: Snakebite incidence and healthcare-seeking behaviors in Eastern Province, Rwanda: A cross-sectional study
Source: PLoS Negl Trop Dis. 2024 Aug 21;18(8):e0012378. doi: 10.1371/journal.pntd.0012378 (PMC11338457; doi:10.1371/journal.pntd.0012378)
Supplement: S5 Appendix — (DOC) [file pntd.0012378.s005.doc]

**Snakebite incidence and healthcare-seeking behavior in Eastern Province, Rwanda: A cross-sectional study**

Dieudonne Hakizimana^1,2^*, Lauren E. MacDonald^3^, Happy Tahirih Kampire^4^; Mihigo Bonaventure^4^; Mahlet Tadesse^4^, Elijah Murara^4^; Leila Dusabe^4^, Leandre Ishema^4^, Janna M. Schurer^4,5^*

**S5 Appendix Quality Control Mechanisms and Study Tools**

All tools were translated into Kinyarwanda, the local language. We developed training materials for Community Health Workers (CHWs). For the household questionnaire filled out by CHWs, we created a Standard Operating Procedure (SOP) detailing how each section should be completed. We also provided a filled-out example questionnaire, reflecting all possible scenarios.

During data collection, forms were reviewed as they were submitted to the office. If an issue was identified (e.g., incompleteness, missing information about all household members), data collectors immediately contacted the CHW in charge and requested a re-collection of the data.

For the health-seeking behavior survey, data collectors were trained to first confirm if the victim was bitten by a snake in 2020 before proceeding with the survey. Each status was recorded on an Excel sheet (refer to the SBE Interview Log for an example).

**Attached Forms:**

- Household questionnaire filled out by the CHWs
- Guide for CHWs on how to fill out the Household questionnaire
- Example of a filled-out Household questionnaire
- Health-seeking behavior questionnaire (both English and Kinyarwanda)
- The SBE Victims Interview Monitoring Log

**Figure A: Household questionnaire filled out by the CHWs**

**Figure B: Guide for CHWs on how to fill out the Household questionnaire**

**Figure C: Example of a filled-out Household questionnaire**

Health-seeking behavior questionnaire (both English and Kinyarwanda)

**Snakebite envenomation in Rwanda: A One Health assessment of incidence, demographics, and care-seeking behaviors**

*{Thank you for agreeing to participate in our study. My name is __________ and I am a researcher working with the University of Global Health Equity in Rwanda. Our research team is interested in assessing care seeking behavior among people bitten by snakes in Eastern Province. This information will help us to advise the government on how to best support snakebite patients. If you agree to participate, we anticipate that the survey will take approximately 10 minutes to complete.}*

**If the participant is willing, take them through informed consent.*

*Date of interview: ____________________*

*District: _____________________*

*Sector: _____________________*

*Cell: _____________________*

*Village: _____________________*

| *Demographics (*at the time of the bite)* | |
| --- | --- |
| 1) What is your age? | ______________ years |
| 2) What is your sex? | 🞎 Female  🞎 Male  🞎 Prefer not to say |
| 3) What is the highest education degree that you have obtained? | 🞎 None  🞎 Primary  🞎 Secondary  🞎 Bachelors  🞎 Other, please specify: ________________ |
| ) What is your primary occupation? | 🞎 Unemployed  🞎 Homemaker  🞎 Farmer/cultivator  🞎 Shopkeeper  🞎 Teacher  🞎 Other: _______________ |
| ) What is your ubudehe category? | 🞎 1  🞎 2  🞎 3  🞎 4 |
| ) Did you have health insurance in 2020? | 🞎 Yes  🞎 No |
| *We would now like to ask you some questions about your snakebite experience. You are free to skip any questions or to stop the interview if you are uncomfortable.* | |
| ) How many times were you bitten^1^ by a snake in 2020? | 🞎 1x  🞎 2x  🞎 Other: _____________________ |
| ) During which month did the snakebite occur? | Month: _______________________ |
| ) In which cell did the snakebite occur? | Cell:___________________________ |
| ) Can you describe what you were doing when you were bitten? | 🞎 Cultivating  🞎 Herding animals  🞎 Working inside house  🞎 Working outside house  🞎 Sleeping inside house  🞎 Sleeping outside house  🞎 Walking/biking on the road  🞎 Other: __________________ |
| ) Did the snakebite occur during the day or night? (Day: 6 am-6 pm; night: 6 pm-6 am) | 🞎 Day  🞎 Night  🞎 I don’t know |
| ) How did you know that you were bitten by a snake? | 🞎 Participant saw the snake bite or spit  🞎 Fang marks were visible  🞎 Physician diagnosis  🞎 Other: __________________ |
| ) Did anyone identify the snake? If yes, please name/describe the snake. | 🞎 Yes: _____________________________  🞎 No |
| ) What part of your body was bitten? (Check all that apply) | 🞎 Foot  🞎 Leg  🞎 Hand  🞎 Arm  🞎 Torso  🞎 Head/face/neck  🞎 Other: _________________ |
| What symptoms did you experience? (Check all that apply) | 🞎 None  🞎 Pain  🞎 Inflammation  🞎 Uncontrolled bleeding at the bite site  🞎 Difficulty breathing  🞎 Sweating  🞎 Lethargy  🞎 Nausea and/or vomiting  🞎 Dizziness  🞎 Numbness  🞎 Paralysis  🞎 Loss of consciousness  🞎 Blindness  🞎 Other: __________________________ |
| ) Did anyone kill the snake? | 🞎 Yes  🞎 No  🞎 I don’t know |
| *We would now like to ask you some questions about your healthcare experiences (*ask questions for most recent bite only)* | |
| When the bite first occurred, did you perform any of the following? (Check all that apply) | 🞎 Tourniquet  🞎 Burning  🞎 Cutting  🞎 Sucking out venom  🞎 Herbal drinks  🞎 Herbal ointments/salves  🞎 Black stone  🞎 Other: _______________________ |
| ) Who did you first approach for treatment?  (Check one) | 🞎 Pharmacist  🞎 Traditional Healer  🞎 Community Health Worker  🞎 Health Center/Post  🞎 Hospital  🞎 Other: ______________________ |
| ) Why was this your first choice? (Check one) | 🞎 Cost  🞎 Trust  🞎 Proximity  🞎 Other: _____________________ |
| ) After your first choice, did you seek care from anyone else? (Check all that apply) | 🞎 No  🞎 Pharmacist  🞎 Traditional Healer  🞎 Community Health Worker  🞎 Health Center/Post  🞎 Hospital  🞎 Other: ______________________ |
| ) If yes, why did you seek care from more than one provider? | 🞎 First provider did not resolve the problem  🞎 First provider was unavailable  🞎 Referral  🞎 Other: __________________ |
| What form(s) of transportation did you use to obtain care?  (Check all that apply) | 🞎 Walk  🞎 Carried by a person/people  🞎 Bicycle  🞎 Motorbike  🞎 Car  🞎 Bus  🞎 Other: ________________ |
| If patient sought care from a pharmacist,   1. What treatment did you receive?     (b) Please rate your level of satisfaction with the care you received | (a)  🞎 Tourniquet  🞎 Burning  🞎 Cutting  🞎 Sucking out venom  🞎 Pain relief  🞎 Anti-swelling  🞎 Antivenom  🞎 Black stone  🞎 Referral  🞎 Other: ____________________  🞎 Unsure  🞎 No treatment  (b)  🞎 Very satisfied  🞎 Someone satisfied  🞎 Not satisfied  🞎 Prefer not to answer |
| If patient sought care from a traditional healer,  (a) What treatment did you receive?    (b) Please rate your level of satisfaction with the care you received | (a)  🞎 Tourniquet  🞎 Burning  🞎 Cutting  🞎 Sucking out venom  🞎 Herbal drinks  🞎 Herbal ointments/salves  🞎 Black stone  🞎 Referral  🞎 Other: ____________________  🞎 Unsure  🞎 No treatment  (b)  🞎 Very satisfied  🞎 Someone satisfied  🞎 Not satisfied  🞎 Prefer not to answer |
| If patient sought care from a CHW,  (a) What treatment did you receive?  (b) Please rate your level of satisfaction with the care you received | (a)  🞎 Tourniquet  🞎 Burning  🞎 Cutting  🞎 Sucking out venom  🞎 Pain relief  🞎 Anti-swelling  🞎 Antivenom  🞎 Black stone  🞎 Referral  🞎 Other: ____________________  🞎 Unsure  🞎 No treatment  (b)  🞎 Very satisfied  🞎 Someone satisfied  🞎 Not satisfied  🞎 Prefer not to answer |
| If patient sought care from a health post/center,  (a) What treatment did you receive?  (b) Please rate your level of satisfaction with the care you received | (a)  🞎 Tourniquet  🞎 Burning  🞎 Cutting  🞎 Sucking out venom  🞎 Pain relief  🞎 Anti-swelling  🞎 Antivenom  🞎 Black stone  🞎 Referral  🞎 Other: ____________________  🞎 Unsure  🞎 No treatment  (b)  🞎 Very satisfied  🞎 Someone satisfied  🞎 Not satisfied  🞎 Prefer not to answer |
| If patient sought care from a hospital,  (a) What treatment did you receive  (b) Please rate your level of satisfaction with the care you received at the health facility | (a)  🞎 Tourniquet  🞎 Burning  🞎 Cutting  🞎 Sucking out venom  🞎 Pain relief  🞎 Anti-swelling  🞎 Antivenom  🞎 Black stone  🞎 Referral  🞎 Other: ____________________  🞎 Unsure  🞎 No treatment  (b)  🞎 Very satisfied  🞎 Someone satisfied  🞎 Not satisfied  🞎 Prefer not to answer |
| ) If patient sought care from a hospital, what was the time interval between being bitten and getting to a hospital?  (*This includes the time for referrals from other health facilities) | # __________________________minutes  🞎 Unsure |
| ) If participant sought care from a hospital, what was the time interval between arriving at the hospital and being examined by a physician? | # ________________________minutes  🞎 Unsure |
| ) If participant went to a hospital, did they experience any delays in receiving care? | 🞎 Yes  🞎 No  🞎 I don’t know |
| If yes, what were the reasons for delay? | 🞎 The hospital had many other patients  🞎 Patient did not have required documentation  🞎 Issues with payment  🞎 The hospital did not have drugs/supplies  🞎 Physician unavailable  🞎 I don’t know  🞎 Other: ___________________________ |
| ) How many days did it take to recover after being bitten? | 🞎 0 days  🞎 1 day  🞎 2 days  🞎 Other: __________________  🞎 Patient died |
| ) How did you pay the treatment costs?  (Check all that apply) | 🞎 N/A (there were no costs)  🞎 Savings  🞎 Loan  🞎 Donation (e.g. family member, friend)  🞎 Sold livestock  🞎 Sold property  🞎 Health insurance  🞎 I don’t know  🞎 Other |
| ) Which statement best describes your current health status? | 🞎 I fully recovered from the snakebite  🞎 I partially recovered and have minor ongoing physical problems  🞎 I have serious ongoing physical disabilities  🞎 Patient died |
| ) Did your experience any other consequences due to snakebite?  (check all that apply) | 🞎 No  🞎 Food insecurity  🞎 Loss of income  🞎 Interrupted school/training  🞎 Divorce/separation from spouse  🞎 Physical disability, including pain, limited mobility  🞎 Fear/anxiety related to snakes  🞎 Other _________________________ |
| ) In your opinion, who provides the best quality care for snakebite? | 🞎 Pharmacist  🞎 Traditional Healer  🞎 Community Health Worker  🞎 Health Center/Post  🞎 Hospital  🞎 Other: ______________________ |
| ) Were you ever been bitten by a snake before 2020? If yes, how many times? | 🞎 Yes: # bites:_______________  🞎 No |
| ) Has any other member of your household ever been bitten by a snake? | 🞎 Yes  🞎 No |
| ) What animals did your household own in 2020?  (Check all that apply) | 🞎 None  🞎 Cat  🞎 Dog  🞎 Goat  🞎 Chicken  🞎 Cattle  🞎 Swine  🞎 Other |
| ) Were any of your animals bitten by snakes in 2020? | 🞎 Yes  🞎 No |
| ) Have any of your animals ever been bitten by a snake? | 🞎 Yes  🞎 No |
| ) Is there anything you do to protect yourself snakebites? If yes, please describe. | 🞎 Yes: _______________________________  🞎 No |
| ) When walking at night, do you carry a torch? | 🞎 Never  🞎 Sometimes  🞎 Always |
| ) Do you sleep under a mosquito net? | 🞎 Never  🞎 Sometimes  🞎 Always |
| ) Is your house secure from snakes entering? | 🞎 Yes  🞎 No |
| ) Is your house well lighted at night? | 🞎 Yes  🞎 No |
| ) Is there anything you do to control rodents in your house? If yes, what? | 🞎 Yes: ________________________  🞎 No |

^1^Snakebite envenomation includes any incidence of physical bite or venom being spat at victim

**Snakebite envenomation in Rwanda: A One Health assessment of incidence, demographics, and care-seeking behaviors**

*{Urakoze kwemera kugira uruhare muri ubu bushakashatsi. Amazina yanjye ni __________ nkaba ndi mu itsinda rya Kaminuka mpuzamahanga y’ubuzima rusange yo mu Rwanda (UGHE, Rwanda). Turifuza kumenya uburyo abaribwa n’inzoka babona ubuvuzi mu mu ntara y’iburasirazuba.* Ibyo bizadufasha gutanga ubujyanama kuri leta ku ngamba zo gufasha abantu baribwa n’inzoga. Mu gihe wemeye kugira uruhare, turagirana ikiganiro gushobora gutwara nk’iminota 10.}

** Mugihe uwitabiriye ubushakashatsi abishaka, musomere ibikubiye mu rupapuro rukubiyemo amakuru no kwemera kugira uruhare mu bushakashatsi ku bushake*

*Italiki y’ikiganiro: ____________________*

*Akarere: _____________________*

*Umurenge: _____________________*

*Akagali: _____________________*

*Umudugudu: _____________________*

| Imyirondoro/Irangamimerere *(*Ku gihe cyo kuribwa n’inzoka)* | |
| --- | --- |
| 1) Ufite imyaka ingahe? | ______________ imyaka |
| 2) Igitsina? | 🞎 Umugore  🞎 Umugabo  🞎 Sinshaka kubivuga |
| 3) Wize amashuri angahe? Ayo warangije (hitamo kimwe) | 🞎 Ntayo  🞎 Amashuri abanza  🞎 Amashuri yisumbuye  🞎 Kaminuza  🞎 Iyandi (yavuge) __________ |
| 4) Ese ukora iki kigutunze? (Hitamo kimwe cy’ingenzi) | 🞎 Ntakazi mfite  🞎 Umukozi wo mu rugo  🞎 Umworozi/Umuhinzi  🞎 Umucuruzi  🞎 Umwarimu  🞎 ibindi: _______________ |
| 5) Uri mu kihe cyiciro cy’ibudehe? (Hitamo kimwe) | 🞎 1  🞎 2  🞎 3  🞎 4 |
| 6) Ese wari ufite ubwisungane mukwivuza mu mwaka wa 2020? | 🞎 Yego  🞎 Oya |
| *Turashaka kukubaza ibibazo bijyanye n’uburyo byagenze ubwo waribwaga n’inzoko. Ubufite uburenganzira bwo kuba wasimbuka ikibazo icyo aricyo cyose cyangwa se guhagarika ikiganiro mu gihe waba wumva utamerewe neza.* | |
| Ese ni inshuro zingahe waba warariwe n’inzoka^1^ mu mwaka wa 2020 | 🞎 Inshuro 1  🞎 Inshuro 2  🞎 Ibindi: _____________________ |
| ) Ese inzoka yakuriye mu kuhe kwezi? | Ukwezi: _______________________ |
| ) Ese waririwe n’inzoka mu kahe kagali? | Cell:___________________________ |
| ) Ese inzoko yakuriye uri gukora iki? | 🞎 Narindi guhinga  🞎 Narindagiye amatungo  🞎 Narindi gukora imirimo mu nzu imbere  🞎 Narindi gukora imirimo mu hanze y’inzu  🞎 Narindyamye mu nzu imbere  🞎 Narindyamye hanze y’inzu  🞎 Narindi kugenda munzira  🞎 Ibindi: __________________ |
| ) Ese inzoka yakuriye nijoro cyangwa ku manywa Did the snakebite occur during the day or night? (Amanywa: kuva saa kumi n’ebyiri za mu gitondo kugera saa kumi n’ebyiri za nimugoroba; ijoro: kuva saa kumi n’ebyiri za nimugoroba kugera saa kumi n’ebyiri za mugitondo) | 🞎 Ku manywa  🞎 Ijoro  🞎 Simbizi |
| Ese wamenye gute ko wariwe n’inzoka? | 🞎 Hari umuntu wabibonye  🞎 Amenyo yaho yariye yaragaragaraga  🞎 Byemejwe n’umuganga  🞎 Ibindi:_________________________ |
| ) Ese haba hari uwabonye iyo nzoka? Niba ari yego, vuga uko iyo nzoka yari imeze | 🞎 Yego: _____________________________  🞎 Oya |
| ) Ese ni ku kige gice cy’umubiri inzoka yakuriyeho? (Hitamo byose bishoboka) | 🞎 Ikirenge  🞎 Ukuguru  🞎 Ikiganza  🞎 Ukuboko  🞎 Ku kiganza  🞎 Umutwe/Mu maso/Ku ijosi  🞎 Ibindi: _________________ |
| Ese wagize ibihe bimenyetso ? (Hitamo byose bishoboka) | 🞎 Ntacyo  🞎 Kubabara  🞎 Kubyimba  🞎 Kuvirirana bidahagarara aho yariye  🞎 Guhumeka bigoranye/nabi  🞎 Kubira ibyunzwe  🞎 Gucika intege  🞎 Iseseme no/cyangwa kuruka  🞎 Kuzengerezwa  🞎 Ibinya  🞎 Pararisi  🞎 Guta ubwenge  🞎 Guhuma  🞎 Ibindi: __________________________ |
| ) Ese haba hari uwishe iyo nzoka? | 🞎 Yego  🞎 Oya  🞎 Simbizi |
| *Tugiye kukubaza ibibazo bijyanye n’uburyo wabonye ubuvuzi nyuma yo kuribwa (*Ibi bibazo birabazwa gusa ku nshuro iheruka yariweho n’inzoka)* | |
| Ese inzoka imaze kukurya, waba warakoze bimwe muri ibi bikurikira” (Hitamo byose bishoboka) | 🞎 Kuzirika ahariwe  🞎 Gutwika  🞎 Gukeba  🞎 Gukurura/kuvoma ubumara  🞎 Imiti y’amazi y’ibyatsi  🞎 Imiti y’’amavuta y’ibyatsi  🞎 Gukoresha akabuye k’umukara  🞎 Ibindi: ______________________ |
| ) Ninde muvuzi/nihe bwa mbere wasabye ubufasha (hitamo kimwe) | 🞎 Kuri farumasi  🞎 Umuvuzi gakondo  🞎 Umujyana w’ubuzima  🞎 Ikigo nderabuzima/Ikigo cy’ibanze  🞎 Ibitaro  🞎 Ahandi: ______________________ |
| ) Ese kuki ariho hantu ha mbere wahisemo? (hitamo kimwe) | 🞎 Igiciro/Ikiguzi  🞎 Niho nizera  🞎 Niho hanyegereye  🞎 Ibindi: _____________________ |
| ) Ese uretse ahambere wavuze, haba hari ahandi washatse ubuvuzi nyuma yaho ha mbere? (Hitamo byose bishoboka) | 🞎 Ntaho  🞎 Kuri farumasi  🞎 Umuvuzi gakondo  🞎 Umujyana w’ubuzima  🞎 Ikigo nderabuzima/Ikigo cy’ibanze  🞎 Ibitaro  🞎 Ahandi: ______________________ |
| ) Niba ari yego, ese kuki warashatse ubufasha ahantu harenze hamwe? | 🞎 Uwambere ntabwo yabashije kumfasha/gukemura ikibazo  🞎 Ahambere nasanze batakoze/badahari  🞎 Banyohereje ahandi  🞎 Ibindi: __________________ |
| Ese ni ubuhe buryo wakoresheje ngo ugere aho wivuje? (Hitamo byose bishoboka) | 🞎 Kugenda n’amaguru  🞎 Natwawe n’abantu mu maboko  🞎 Nagiye ku igare  🞎 Nagiye kuri moto  🞎 Imodoka  🞎 Bisi cg Imodoka rusange itwara abagenzi  🞎 Ubundi buryo: ________________ |
| Niba umurwayi yarashatse ubufasha/yaragiye kwivuza kuri farumasi,  a) Ni ubuhe buvuzi bamuhaye/Bamukoreye iki? (Hitamo byose bishoboka)  (b) Ese ni gute wishimiye/wanyuzwe n’ubufasha/ubuvuzi wahawe? | (a) Ibyo bagukoreye:  🞎 Kuzirika ahariwe  🞎 Gutwika  🞎 Gukeba  🞎 Gukurura/kuvoma ubumara  🞎 Imiti igabanya uburibwe  🞎 Imiti igabanya kubyimba  🞎 Imiti irwanya ubumara  🞎 Gukoresha akabuye k’umukara  🞎 kukohereza ahandi  🞎 Ibindi: ______________________  🞎 Simbizi  🞎 Ntacyo nakorewe  (b)  🞎 Bwaranshimishije cyane  🞎 Bwaranshimishije gahoro  🞎 Ntabwo bwanshimishije  🞎 Sinshaka kubivugaho/kubisubiza |
| Niba umurwayi yarashatse ubufasha/yaragiye kwivuza ku muvuzi wa gakondo,  a) Ni ubuhe buvuzi bamuhaye/Bamukoreye iki? (Hitamo byose bishoboka)    (b) Ese ni gute wishimiye/wanyuzwe n’ubufasha/ubuvuzi wahawe? | (a) Ibyo bagukoreye:  🞎 Kuzirika ahariwe  🞎 Gutwika  🞎 Gukeba  🞎 Gukurura/kuvoma ubumara  🞎 Imiti y’amazi y’ibyatsi  🞎 Imiti y’’amavuta y’ibyatsi  🞎 Gukoresha akabuye k’umukara  🞎 kukohereza ahandi  🞎 Ibindi: ______________________  🞎 Simbizi  🞎 Ntacyo nakorewe  🞎 Bwaranshimishije cyane  🞎 Bwaranshimishije gahoro  🞎 Ntabwo bwanshimishije  🞎 Sinshaka kubivugaho/kubisubiza |
| Niba umurwayi yarashatse ubufasha/yaragiye kwivuza ku mujyanama w’ubuzima,  a) Ni ubuhe buvuzi bamuhaye/Bamukoreye iki? (Hitamo byose bishoboka)    (b) Ese ni gute wishimiye/wanyuzwe n’ubufasha/ubuvuzi wahawe? | (a) Ibyo bagukoreye:  🞎 Kuzirika ahariwe  🞎 Gutwika  🞎 Gukeba  🞎 Gukurura/kuvoma ubumara  🞎 Imiti igabanya uburibwe  🞎 Imiti igabanya kubyimba  🞎 Imiti irwanya ubumara  🞎 Gukoresha akabuye k’umukara  🞎 kukohereza ahandi  🞎 Ibindi: ______________________  🞎 Simbizi  🞎 Ntacyo nakorewe  (b)  🞎 Bwaranshimishije cyane  🞎 Bwaranshimishije gahoro  🞎 Ntabwo bwanshimishije  🞎 Sinshaka kubivugaho/kubisubiza |
| Niba umurwayi yarashatse ubufasha/yaragiye kwivuza ku kigonderabuzima/ikigonderabuzima cy’ibaze (poste de sante)  a) Ni ubuhe buvuzi bamuhaye/Bamukoreye iki? (Hitamo byose bishoboka)    (b) Ese ni gute wishimiye/wanyuzwe n’ubufasha/ubuvuzi wahawe? | (a) Ibyo bagukoreye:  🞎 Kuzirika ahariwe  🞎 Gutwika  🞎 Gukeba  🞎 Gukurura/kuvoma ubumara  🞎 Imiti igabanya uburibwe  🞎 Imiti igabanya kubyimba  🞎 Imiti irwanya ubumara  🞎 Gukoresha akabuye k’umukara  🞎 kukohereza ahandi  🞎 Ibindi: ______________________  🞎 Simbizi  🞎 Ntacyo nakorewe  (b)  🞎 Bwaranshimishije cyane  🞎 Bwaranshimishije gahoro  🞎 Ntabwo bwanshimishije  🞎 Sinshaka kubivugaho/kubisubiza |
| Niba umurwayi yarashatse ubufasha/yaragiye kwivuza ku bitaro  a) Ni ubuhe buvuzi bamuhaye/Bamukoreye iki? (Hitamo byose bishoboka)    (b) Ese ni gute wishimiye/wanyuzwe n’ubufasha/ubuvuzi wahawe? | (a) Ibyo bagukoreye:  🞎 Kuzirika ahariwe  🞎 Gutwika  🞎 Gukeba  🞎 Gukurura/kuvoma ubumara  🞎 Imiti igabanya uburibwe  🞎 Imiti igabanya kubyimba  🞎 Imiti irwanya ubumara  🞎 Gukoresha akabuye k’umukara  🞎 kukohereza ahandi  🞎 Ibindi: ______________________  🞎 Simbizi  🞎 Ntacyo nakorewe  (b)  🞎 Bwaranshimishije cyane  🞎 Bwaranshimishije gahoro  🞎 Ntabwo bwanshimishije  🞎 Sinshaka kubivugaho/kubisubiza |
| ) Niba umurwayi yaragiye kwivuza ku bitaro, ese byatwaye igihe kingana iki kuva inzoka imurumye kugera ageze ku bitaro? (*Habariwemo n’igihe cyo kumwohereza ava mu yandi mavuriro atandukanye) | # __________________________iminota  🞎 Simbizi neza |
| ) Niba umurwayi yaragiye kwivuza ku bitaro, ese byatwaye igihe kingana iki kuva ageze ku bitaro kugera asuzumwe na muganga | # ________________________iminota  🞎 Simbizi neza |
| ) Niba umurwayi yaragiye kwivuza ku bitaro, ese yaba yaratinze kuvurwa? | 🞎 Yego  🞎 Oya  🞎 Siimbizi |
| Niba ari yego, byaba byaratewe n’iki ngo atinde kuvurwa? | 🞎 Hari abanda barwayi benshi ku bitaro  🞎 Umurwayi ntabwo yari afite impampuro zose zisabwa  🞎 Ibibazo bijyanye no kwishyura  🞎 Ibitaro ntibyari bifite ibikoresho n’imit bisabwa  🞎 Umuganga ntabwo yari ahari/yabonekaga  🞎 Simbizi  🞎 Ibindi: ___________________________ |
| ) Ese byatwaye igihe kingana iki ngo ukire nyuma yo kurumwa n’inzoka? | 🞎 Munsi y’umunsi 1  🞎 Umunsi 1  🞎 Iminsi 2  🞎 Ibindi: __________________  🞎 Umurwayi yarapfuye |
| ) Ese wishyuye ute ubuvuzi wahawe?(Hitamo byose bishoboka) | 🞎 Singombwa (Ntakiguzi)  🞎 Ayo nari narizigamiye  🞎 Nafashe inguzanyo  🞎 Ubufasha (e.g. abo mu muryango, inshuti)  🞎 Nagurishije itungo  🞎 Nagurishije bimwe mubyo nari ntunze  🞎 Ubwisungane mu kwivuza  🞎 Simbizi  🞎 Ibindi |
| ) Ese ubu wumva umeze ute mu buzima bwawe uyu munsi? | 🞎 Ndumva narakize neza ntakibazo  🞎 Nabwo nakize neza, numva narasigaranye ibibazo/ubumuga ariko budakanganye  🞎 Mfite ubumuga bukomeye bwaturutse ku kuribwa n’inzoka  🞎 Umurwayi yarapfuye |
| ) Ese haba hari izindi ngaruka zaba zaraturutse ku kuribwa n’inzoka? (Hitamo byose bishoboka) | 🞎 Oya ntazo  🞎 Kubura ibyo kurya  🞎 Guhomba umutungo/inyungu ninjizaga  🞎 Guhagarara kw’ishuri/amahugurwa  🞎 Gutanduka nuwo twashakanye  🞎 Ubumuga kumubiri harimo kubabara, kudakora neza kw’ingingo  🞎 Ubwoba/guhangayika bituruka ku nzoka  🞎 Ibindi _________________________ |
| ) Ese muri rusange, ubona arinde cyangwa uruhe rwego rutanga ubuvuzi bwiza ku bantu bariwe n’inzoka? | 🞎 Kuri farumasi  🞎 Umuvuzi gakondo  🞎 Umujyana w’ubuzima  🞎 Ikigo nderabuzima/Ikigo cy’ibanze  🞎 Ibitaro  🞎 Ahandi: ______________________ |
| ) Ese waba wari warigeze uribwa n’inzoka mbere y’umwaka wa 2020? Niba ari yego, ni inshuri zingahe? | 🞎 Yego: # inshuro:_______________  🞎 Oya |
| ) Ese haba hari undi wo mu muryango wawe mubana waba warigeze aribwa n’inzoka? | 🞎 Yego  🞎 No |
| ) Ese ni ayahe matungo mwaba mwaratunze mu mwaka wa?  (Hitamo byose bishoboka) | 🞎 Ntayo  🞎 Injangwe  🞎 Imbwa  🞎 Ihene  🞎 Inkoko  🞎 Inka  🞎 Ingurube  🞎 Ayandi |
| ) Ese haba hari itungo na rimwe ryaba ryarigeze riribwa n’inzoka mu mwaka wa 2020? | 🞎 Yego  🞎 Oya |
| ) Ese haba hari itungo na rimwe ryaba ryarigeze riribwa n’inzoka haba na mbere y’u mwaka wa 2020? | 🞎 Yego  🞎 Oya |
| ) Ese haba hari icyo ukora ngo wirinde kuribwa n’inzoka? Niba ari yego, watubwira ibyo ukora muri macye. | 🞎 Yego: _______________________________  🞎 Oya |
| ) Ese iyo ugenda nijoro, ugendana itoroshi yo kumurika? | 🞎 Nta na rimwe  🞎 Rimwe na rimwe  🞎 Iteka ryose |
| ) Ese urara munzitiramibu? | 🞎 Nta na rimwe  🞎 Rimwe na rimwe  🞎 Iteka ryose |
| ) Ese inzu yawe ifite ubwirinzi butuma inzoka zitakwinjiramo? | 🞎 Yego  🞎 Oya |
| ) Ese inzu yawe iba ifite urumuri ruhagije nijoro? | 🞎 Yego  🞎 Oya |
| ) Ese haba hari icyo ukora kugirango wirinde izindi nyamaswa nk’imbeba munzu? Niba ari yego, waba ukora iki? | 🞎 Yego: ________________________  🞎 Oya |

^1^Kuribwa n’inzoka harimo gushingwa amenyo n’inzoka cyangwa se gucirwa amacandwe n’inzoka

The SBE Victims Interview Monitoring Log
